# Medical Items

**Published:** 1894-07

**Authors:** 


### *MEDICAL ITEMS.*

---

THE new building of the New York Post-graduate Medical School Hospital was formally opened May 8th.

---

MR. CHARLES HARRISON is the newly-elected Provost of the University of Pennsylvania to succeed Professor William Pepper, resigned.

---

IT is said that the frequent use of *methylbenzonethoxyethyletetrahydrophyridinecarboxylate* will establish a habit. If the habit is as difficult to cure as the name is to pronounce and remember, we suppose no one has ever had it and survived.

---

WE learn that internal dissensions in the Medical Association of Mississippi have necessitated its rehabilitation One hundred and twenty-five prominent Mississippi doctors met in Jackson, May 1st, and organized the Medical and Surgical Society of Mississippi.

---

DR. EMORY LANPHEAR, for many years editor of the *Kansas City Medical Index*, has resigned the chair of Operative Surgery and Clinical Surgery in the Kansas City Medical College, and has removed to St. Louis. He makes the change in order to become Professor of Surgery in the reorganized St. Louis College of Physicians and Surgeons, one of the oldest and strongest medical schools in the West.

---

THE College of Physicians and Surgeons of St. Louis has lately made a move which might be emulated with advantage by some of the other ten colleges in that city. The entire faculty resigned and called for a new deal. The work of rehabilitation is now going on. There are too many schools in St. Louis. If the number were reduced by half medical education in this Western medical center would be vastly improved.

---

DOKTORS are not all quaks; you have got wrong noshuns about this. Doktors, lawyers and ministers hav a hard row to ho; they hav to deal with kredulity, knavery and fears of the people—three of the most difficult traits in human natur tew handle. If i was a doktor and understood my bizzness, i should doktor my pashunts, and let the disease take care of itself. More folks are cured this way than enny other.—*Josh Billings.*

---

A STORY is told of a good woman who joined the Methodist Church, but after a while she became dissatisfied and went to a Baptist pastor, and he immersed her and she joined the Baptist Church. After a while she came tearfully and sorrowfully to see her Baptist parson, and she said, "Oh, pastor! pastor!"

He said, "Why, my good sister, what's the matter now? You've been sprinkled, and you've been immersed. What else do you want?"

"Oh, pastor!" she said; "oh, pastor! I want to be circumcised."
—*The Chironian.*

---

A GRIM VIEW OF IT.—The death of an ossified man in Tennessee is reported. He died hard.—*Chicago Tribune.* This is as bad as the man who swallowed a thermometer and died by degrees; it suggests also the case of the consumptive undertaker who died of a coffin.—*Medical Record.* These remind us of a man who choked while eating an apple and died of appleplexy.—*National Medical Review.* It was in a St. Louis hotel that a Pike county farmer blew out the gas and died from gastritis.—*Meyer Brothers' Druggist.* Not any worse than the man struck by an engine; verdict, died from locomotor attacksia.—*Montreal Pharm. Journ.* Still worse the case of that pie-eat-ing dyspeptic of Tiflis, for he died of piemia, superinduced by typhlitis—*Gaillard's Med. Journal.* The other day a negro in Southern Georgia ate six watermelons. He died of meloncholia.

THE late meeting of the National Association of Railway Surgeons in Galveston is said to have resembled in many respects a ward political meeting. The "ring" was on hand, and in their efforts to control things came near destroying the Association.

We learn that pending the election of officers " all had the floor at one time ; all spoke at once and with power ; and the presiding officer was helpless to preserve order. " Prudent counsels, however, finally prevailed, and after an unbecoming wrangle which consumed much of the Association's time, the regular order of papers was taken up. Many papers announced for presentation could not be read. It was agreed to establish an official journal, to be edited by Dr. J. Harvey Reed, of Columbus, Ohio. The next meeting of the Association will be held in Chicago in 1895. The officers for the coming year are :

Dr. C. W. Thorn, of Toledo, Ohio, President ; A. A. Thompson, of Waxahachie, Texas, Secretary ; Dr. Eugene R. Lewis, of Kansas City, Mo., Treasurer.

---

PROFESSOR DAVID CERNA, of the University of Texas, has been writing some exceedingly interesting papers on Mexican history. One of these is an historical study on the Pilgrimage and Civilization of the Toltecs." These were an agricultural and migratory people, generally believed to have been the " most ancient of the civilized Mexican nations. . . . . They were the first race to disseminate over the virgin regions of the American Continent the true germs of civilization. . . . They represent the most primitive nations of Mexico and embrace the sum of all the knowledge found in the most ancient human history of the American Continent."

Dr. Cerna has also contributed some late articles to the *Tri-State Medical Journal* on " Aztec Medicine." They describe in a very interesting manner the origin and growth of medicine among the Aztecs. Dr. Cerna deserves much credit for his valuable studies upon the civilization and medical knowledge among these ancient peoples.

---

DR. JAS. B. HINKLE died in jail in Americus, June 9th, from a self-administered dose of morphine. The sad circumstances which led to this suicide are very well known. On December 21, 1892, Dr. Hinkle shot and killed Dr. J. B. Worsham on account of some testimony which the latter gave in a lawsuit against him. In this difficulty Dr. Hinkle was aided by his son, also a doctor. After the killing public sentiment was very strong against the Hinkles, and there were threats and fears of mob violence. The case came to trial in January last and continued three weeks. It will be remembered as one of the most noted cases in the criminal annals of Georgia. Dr. Hinkle was found guilty and sentenced to the penitentiary for life. An attempt was made to secure a new trial, but the supreme court refused it. Seeing that his case was hopeless, and no prospect before him but to spend the remainder of his days in prison, Dr. Hinkle swallowed several grains of morphine on the 7th of June, and died two days later. His body was buried in Macon. Dr. Hinkle, Jr., is still in jail awaiting trial.

---

Dr. William T. Briggs, Professor of Surgery in the medical departments of the University of Nashville and Vanderbilt University, died June 13th. He was born in Kentucky, 1828, and when twenty years of age graduated in medicine in the medical department of Transylvania University. In 1852 he was elected Demonstrator of Anatomy in the medical department of the University of Nashville. In 1856 he became Professor of Anatomy and in 1868 he succeeded Dr. Paul F. Eve in the Chair of Surgery, which position he filled with success and honor up to his death. Dr. Briggs was one of the most distinguished Southern surgeons, and an ornament to American surgery. In 1881 he was elected Vice-President of the American Medical Association; in 1885 he was made President of the American Surgical Association, of which he was one of the founders; he was President of the Surgical Section of the International Congress in Washington in 1887; and in 1890 he was elected President of the American Medical Association. In spite of a large practice and his collegiate duties Dr. Briggs found time to write many surgical papers of great value, among them a "History of Surgery in Tennessee."

